# DEPCOD: a tool to detect and visualize co-evolution of protein domains

**DOI:** 10.1093/nar/gkac349

**Published:** 2022-05-10

**Authors:** Fei Ji, Gracia Bonilla, Rustem Krykbaev, Gary Ruvkun, Yuval Tabach, Ruslan I Sadreyev

**Affiliations:** Department of Molecular Biology, Massachusetts General Hospital, Boston, MA, USA; Department of Genetics, Harvard Medical School, Boston, MA, USA; Department of Molecular Biology, Massachusetts General Hospital, Boston, MA, USA; Department of Genetics, Harvard Medical School, Boston, MA, USA; Department of Molecular Biology, Massachusetts General Hospital, Boston, MA, USA; Department of Molecular Biology, Massachusetts General Hospital, Boston, MA, USA; Department of Genetics, Harvard Medical School, Boston, MA, USA; Department of Developmental Biology and Cancer Research, Faculty of Medicine, The Hebrew University of Jerusalem, Ein Kerem 9112102, Israel; Department of Molecular Biology, Massachusetts General Hospital, Boston, MA, USA; Department of Pathology, Massachusetts General Hospital and Harvard Medical School, Boston, MA, USA

## Abstract

Proteins with similar phylogenetic patterns of conservation or loss across evolutionary taxa are strong candidates to work in the same cellular pathways or engage in physical or functional interactions. Our previously published tools implemented our method of normalized phylogenetic sequence profiling to detect functional associations between non-homologous proteins. However, many proteins consist of multiple protein domains subjected to different selective pressures, so using protein domain as the unit of analysis improves the detection of similar phylogenetic patterns. Here we analyze sequence conservation patterns across the whole tree of life for every protein domain from a set of widely studied organisms. The resulting new interactive webserver, DEPCOD (DEtection of Phylogenetically COrrelated Domains), performs searches with either a selected pre-defined protein domain or a user-supplied sequence as a query to detect other domains from the same organism that have similar conservation patterns. Top similarities on two evolutionary scales (the whole tree of life or eukaryotic genomes) are displayed along with known protein interactions and shared complexes, pathway enrichment among the hits, and detailed visualization of sources of detected similarities. DEPCOD reveals functional relationships between often non-homologous domains that could not be detected using whole-protein sequences. The web server is accessible at http://genetics.mgh.harvard.edu/DEPCOD.

## Graphical Abstract

**Graphical Abstract:**
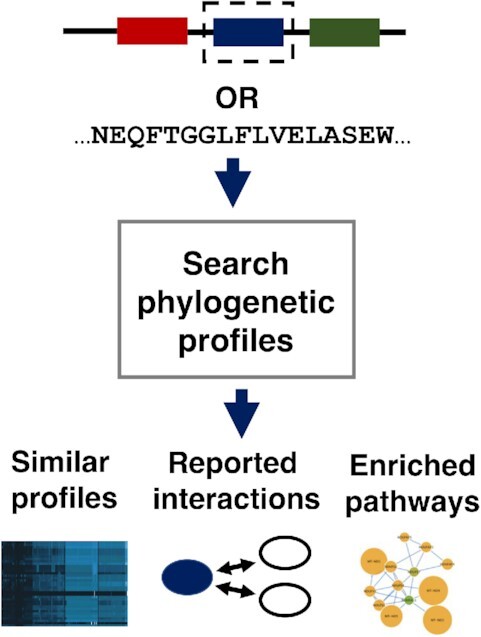
Using pre-defined protein domain or user-supplied sequence as a query, DEPCOD detects other domains with similar phylogenetic profiles, displaying them along with reported protein interactions and pathway enrichment among hits.

## INTRODUCTION

A shared evolutionary history of two proteins across various organisms may suggest similar functions, shared cellular pathways and protein complexes, or functional interactions between these proteins regardless of whether they are homologous to each other (1–6). Our first generation PhyloGene webserver (7), publicly available since 2015, implemented our method of normalized phylogenetic profiling (NPP) of whole-protein sequences, which has been used to detect protein functional associations and predict function of previously uncharacterized proteins, identify new members of metabolic and regulatory pathways, and reveal protein and pathway adaptions of specific organisms (8–17). Proteins that act in the same pathway often have very similar patterns of conservation, retention, or loss of their homologs in particular taxa of organisms, which can be represented in the form of their phylogenetic profiles. As an intensively studied example, a query of one electron transport complex protein will reveal many other complex I proteins with no homology to the query protein but a similar phylogenetic pattern of sequence conservation, instantly revealing functional connections established by 50 years of experimental mitochondrial biochemistry (18). This approach can be applied to less studied pathways as well.

Phylogenetic profiling depends on detecting conservation between individual representatives of the same sequence family in taxonomically distant organisms. However, different domains within the same protein often evolve under different evolutionary constrains and occur in various combinations in different species, especially between taxa at higher levels of taxonomic hierarchy. Protein domains are the functional modules of proteins that can fold, function, and evolve often independently of other domains in the same protein. Variation of particular protein domains or even abrupt changes of domain architecture during evolution may often be due to the relaxation of past functional requirements and changing evolutionary pressures on domain function as organisms specialize for new niches or evolve displacing pathways. These differences of conservation patterns among different domains of the same protein reduce the level of sequence similarity in the analyses of conservation at the level of the whole protein. Therefore, focusing on protein domains as independent evolutionary units should bring more biological relevance and clearer correlations in detecting similar patterns of sequence conservation. Every protein domain in a given genome can be assigned a phylogenetic profile of its relative conservation, variation, or loss based on its sequence similarity to the homologs across hundreds of diverse eukaryotic and prokaryotic species. This phylogenetic pattern can be used to search for non-homologous protein domains with a similar pattern of conservation or loss. This general approach has been discussed and implemented previously, but mainly in the context of domain identification within protein sequences (19–21) and prediction of protein-protein interactions (22). To our knowledge, there are no publicly available web server tools for the similarity detection and visualization of phylogenetic profiles of individual protein domains.

Here, we developed a new interactive web server, DEPCOD (DEtection of Phylogenetically COrrelated Domains), which allows the user to submit a query protein domain from an individual protein in a selected organism and (a) instantly identify the taxa of organisms that have conserved, varied, or lost this domain; (b) detect other protein domains in the same organism that have a correlated pattern of sequence conservation across a wide range of taxonomically diverse species; (c) understand functions of these domains, known physical interactions and shared protein complexes with the query and (d) inspect possible sources and evolutionary relevance of the similarity between their conservation patterns.

This new web server reveals functional relationships between individual domains beyond the detection based on whole-protein sequences. In addition, DEPCOD introduces a combination of methodological and functional features, including: (i) expanded scope of species in evolutionary profiles at two scales: 244 eukaryotic genomes or 506 genomes from all three domains of life: *Eukaryota*, *Bacteria*, and *Archaea*; (ii) incorporation of phylogeny of the searched genomes into the correlation of conservation patterns; (iii) visualization of known BioGRID (23) and hu.MAP (24) interactions and shared protein complexes between the query and the detected hits; (iv) analysis of GO (25,26), KEGG (27,28) and Reactome (29) pathway enrichment among the detected hits and (v) visualization of details and sources of detected patterns similarities (conservation values for individual species, taxonomic trees, links to the information about detected domains and domain families, etc).

## MATERIALS AND METHODS

Using PFAM (30) domain annotation within protein sequences in the genomes of widely studied organisms (*H. sapiens*, *M. musculus*, *D. melanogaster*, *C. elegans*, *S. cerevisae*, *A. thaliana*, *E. coli*, *B. subtilis*), we split the whole sequence of each protein into PFAM domains (30) and parts without detected PFAM homologs.

The resulting sequences were used to generate a domain based normalized phylogenetic profile (NPP) for each domain across a wide range of sequenced genomes in a fashion similar to our previously described approach (7,9). In brief, each domain was used as a query for the BLASTP search in our comprehensive database compiled of all proteins from 506 representative genomes from all three domains of life. Scores of top BLAST hits with moderate to high significance were normalized by the BLAST score of the query to itself, and then transformed into genome-specific *Z*-scores based on the distributions of normalized BLAST scores across a given representative genome. As a result, each domain from the query genomes of model organisms was assigned a NPP, a vector of *Z*-scores for its closest homolog in each of 506 representative target genomes.

To assess the similarity between evolutionary conservation patterns of two domains from the same query genome, their NPPs were compared to each other. As a measure of similarity between profiles, we used Pearson correlation coefficient between two vectors of corresponding *Z*-scores. We chose Pearson *R* over an alternative of Spearman correlation coefficient (a more robust but less sensitive measure of correlation) since in our tests Pearson *R* produced more accurate and functionally relevant results. As DEPCOD profiles are based on a larger number of target genomes, we introduced a new modification into the calculation of Pearson correlation coefficient, which downweighs closely related species among 506 genomes by weighing each target genome proportionally to the number of genomes sampled from the same taxonomic clade in the NCBI taxonomy (31,32). To estimate statistical significance of the resulting profile similarity, we calculated a *Z*-score using the empirical distribution generated by random shuffling of weighted Pearson coefficients across target genomes. Based on extensive manual inspection of DEPCOD hits for multiple queries, we suggest the approximate cutoffs of Pearson *R* >0.6 and significance *Z*-score >4.0 as a combined criterion of a confident profile similarity to the query. To highlight these confident hits, we introduced a separate column in the output heatmap, ‘Correlated and significant’. However, the user is encouraged to inspect the hits beyond this combined criterion, as these domains can sometimes also show functional associations with the query.

For each pre-defined standard domain from a model genome, the NPP is pre-computed and stored along with the top correlated NPPs from the same genome and the corresponding Pearson correlation coefficients and their statistical significance. If the user selects the query from the menu of pre-defined domains in the model genome of choice, then the query NPP and top hits are quickly retrieved from the pre-computed set of domain NPPs. If the user chooses to submit an amino acid sequence as a query, then query NPP is calculated by first running BLASTP with the user-supplied sequence against the set of proteins in all target genomes. Depending of the user's choice, these calculations can be performed across 244 eukaryotic genomes or the full set of 506 species including *Archaea* and *Bacteria*.

To provide the information about known physical protein-protein interactions of each hit with the query, we used the confidence values for interactions from BioGRID (23) and Hu.Map 2.0 (24), as well as predictions of protein complexes from Hu.Map 2.0.

To implement the analysis of functional enrichment among detected hits, we used protein lists for GO biological process level 3 (25,26), KEGG (27,28) and Reactome databases (29). The statistical significance of pathway enrichment is calculated as a hypergeometric *P*-value with Benjamini-Hochberg False Discovery Rate correction for multiple testing. These *P*-values are pre-computed and stored for the standard pre-defined query domains or calculated in real time for the hits based on a user-supplied domain sequence.

To evaluate the accuracy of detection of functional associations, we generated precision/recall curves using protein pairs sharing the same functional pathway as a reference of true associations. We used GO biological process level 3 (25,26), KEGG (27,28), and Reactome databases (29) as three alternative sources of these reference associations. For each protein domain in human genome, top 100 hits with Pearson *R* >0.6 were selected, the resulting hits for all domains were ranked, and precision (TP/(TP + FP)) and recall (TP/(TP + FN)) were calculated for each position *n* in the list, where TP, FP and FN are the numbers of true positives, false positives, and false negatives among top *n* hits in the list.

## RESULTS

### Input and output

DEPCOD webpage (Supplementary Figure S1) includes three main areas. The top area includes buttons for basic information (‘About’), more detailed manual (‘Help’) and selection of major search flavors (‘Eukaryotes’ vs ‘All clades’), which gives the user the choice of evolutionary range across which the phylogenetic profiles are compared (Supplementary Figure S1). ‘All clades’ option corresponds to the construction and comparison of phylogenetic profiles across the whole tree of life (*Eukaryota*, *Bacteria* and *Archaea*), whereas ‘Eukaryotes’ option is focused on eukaryotic species, which would be more relevant for eukaryote-specific query domains or for more detailed analysis of conservation patterns among eukaryotes only.

The area on the left of the webpage (Supplementary Figure S1) includes menus and windows for query submission and defining search parameters. The user can choose the query organism from the set of widely studied species: *H. sapiens*, *M.musculus*, *D. melanogaster*, *C. elegans*, *S. cerevisae* and *A. thaliana* for the search among profiles based on eukaryotic genomes, with the addition of bacterial genomes of *E. coli* and *B. subtilis* for the search among profiles based on the whole tree of life. After the organism is selected, the user can use the autocomplete search menu to quickly choose the query domain from the pre-defined list of protein domains in the given genome. The user can also select the number (50, 100, or 200) of top correlated protein domains to display in the output. Alternatively, the user can provide an arbitrary amino acid sequence as a query, either by pasting this sequence in the window or by uploading a sequence file.

Clicking the Submit button starts the search and generates the output in the main area of the webpage (Supplementary Figure S1), which includes the interactive heatmap of phylogenetic profiles for the top hits, the display of protein function enrichment among these hits, and buttons to save the link to this output page (‘Share’), download the detailed numerical profiles as an Excel or tab-delimited file (‘Download’), or navigate directly to the function enrichment results (‘Gene Set Enrichment’). In the heatmap of top correlated phylogenetic profiles, the main discovery tool, the top row corresponds to the query domain and each row below corresponds to an individual protein domain whose phylogenetic profile is similar to the query. Each column corresponds to an individual species from the selected taxonomic range. Shades of blue denote the normalized BLAST similarity score against the query and white denotes the absence of similarity detected above the BLAST cutoff in the given species. When such absence is observed in multiple related taxa within the taxonomic tree shown above this heatmap, this indicates a stronger variation or a complete loss of the protein domain in that taxonomic group. Apart from more trivial profile similarities between domains from the same protein or protein paralogs when entire proteins co-evolve as single units, there are frequent cases of detecting other non-homologous protein domains with highly correlated phylogenetic profiles. These domains from non-homologous proteins are likely candidates for functional associations with the query.

**Figure 1.**
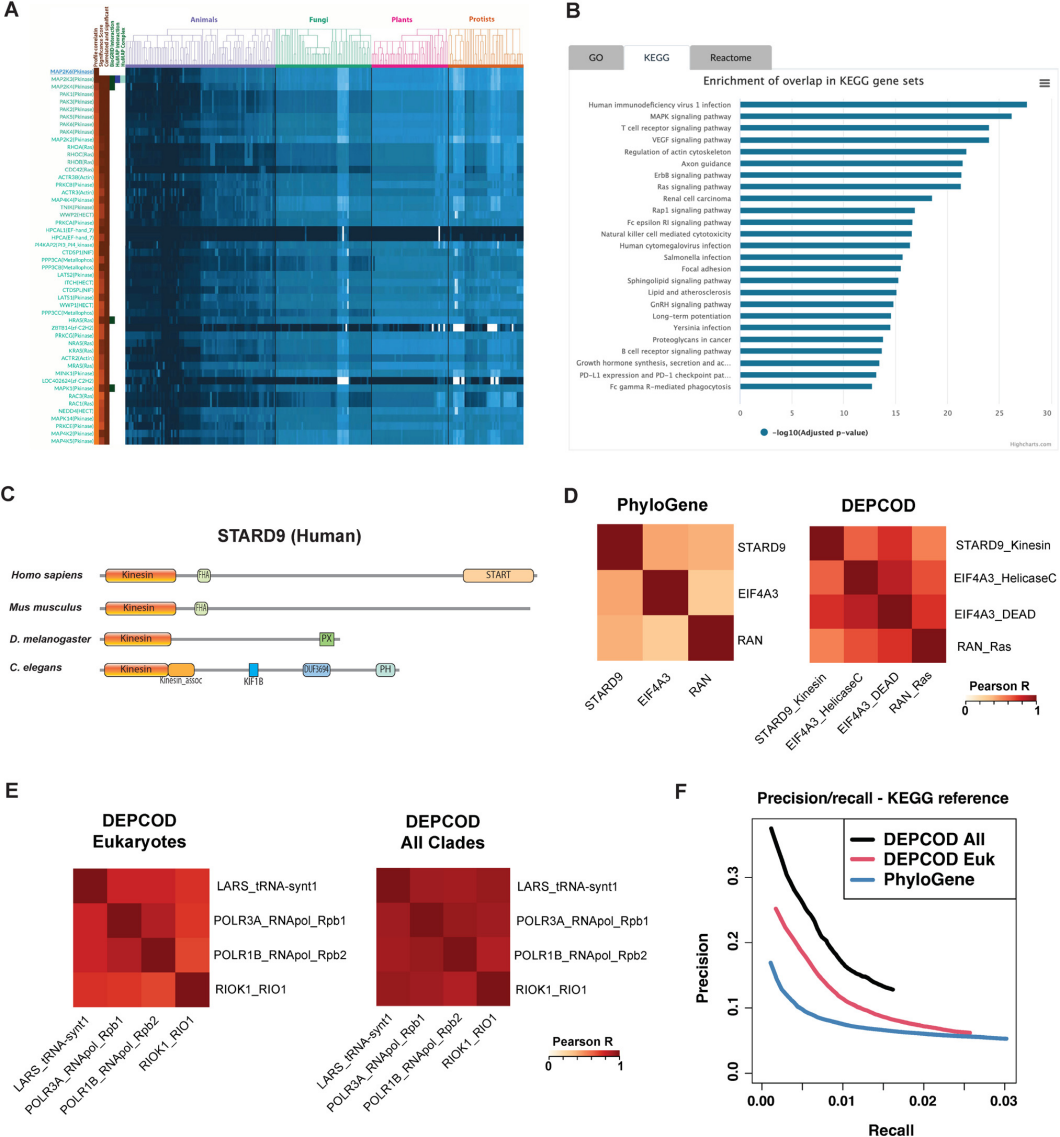
(**A**) Example of DEPCOD output: the heatmap of top eukaryotic phylogenetic profiles most similar to that of Protein Kinase domain from human MAP2K6 protein as a query. Rows, top human domain hits, with query domain on top. Columns, individual species within a chosen evolutionary range (eukaryotes in this example), with taxonomic tree of these genomes shown on top. Hues of blue indicate normalized sequence similarity scores across all species to the human domain. Two yellow-brown columns on the left: Pearson correlation coefficient (left) and the corresponding statistical significance *Z*-score (second left) for the comparison between the given profile and the profile for the query domain (top row). The third column (‘Correlated and significant’) highlights the most confident hits that satisfy the cutoffs of both Pearson *R* and *Z*-score. Next three white-green columns on the left: BioGRID and Hu.Map scores for the physical interactions between the corresponding proteins and Hu.Map score for sharing the same protein complex. (**B**) The barplot of statistical significance of functional enrichment among the top domain hits (-log10 of Benjamini-Hochberg False Discovery Rate) based on functional gene sets from the KEGG database. (**C**) Evolutionary rearrangements of domain architecture between different species reduce the similarity of whole-protein phylogenetic profiles. PFAM domain architecture for human STARD9 protein (UniProt ID Q9P2P6) and corresponding proteins in mouse (Stard9, UniProt ID Q80TF6), fly (Klp98A, UniProt ID Q9VB25), and worm (unc-104, UniProt ID P23678). Kinesin domain at the N terminus is highlighted in orange. Changes in composition of other domains between species obstruct the detection of profile similarity using whole-protein sequences. (**D**) As a result, our previous PhyloGene method based on whole-protein sequences was not able to produce strong correlation estimates between phylogenetic profiles of STARD9 and two functionally related non-homologous proteins EIF4A3 and RAN, whereas DEPCOD detected strong correlation between individual domains of these proteins. Heatmaps of Pearson correlation coefficients for whole-protein sequences (PhyloGene, left) compared to individual domain sequences (DEPCOD, right). (**E**) Example of increased correlation between phylogenetic profiles when these profiles were expanded from eukaryotic species to the whole tree of life. Heatmaps of all-to-all Pearson correlation coefficients between tRNA synthase 1 domain of human LARS protein as a query and domains of functionally associated proteins POLR3A, POLR1B and RIOK1. Phylogenetic profiles based on eukaryotes had only modest correlations (R < 0.5) for most domain pairs (left), which increased to much higher levels when species across the whole tree of life were used (right). (**F**) Precision/recall plots comparing the accuracy of detecting functional protein associations using phylogenetic profiles based on whole proteins (PhyloGene) and on protein domains (DEPCOD). KEGG pathways were used as a benchmarking reference, with the definition of a true positive hit based on sharing the same KEGG pathway with the query. DEPCOD has a higher accuracy than PhyloGene. DEPCOD mode with phylogenetic profiles based on the whole tree of life (DEPCOD All) has a higher accuracy than the mode based on eukaryotes only (DEPCOD Euk).

Examples of DEPCOD output are shown in Figures 1A, B. When DEPCOD is supplied with Kinesin domain from human MAP2K6 protein as a query, it displays the heatmap of top eukaryotic phylogenetic profiles most similar to that of the query (Figure 1A) and the enrichment of functional gene categories among these hits based on three alternative functional gene set databases (Figure 1B). Names of top hits are shown to the left of the heatmap and are ranked by the correlation of their conservation pattern to the query domain shown at the top of the heatmap (Figure 1A). Pearson correlation coefficients (‘Profile correlation’) and *Z*-scores of their statistical significance (‘Significance Score’) are shown by color in two leftmost columns of the heatmap adjacent to the hit names. The third column (‘Correlated and significant’) highlights the most confident hits which satisfy the suggested cutoffs of Pearson *R* > 0.6 and significance *Z*-score > 4.0 as a combined criterion of a confident profile similarity, based on extensive manual inspection of DEPCOD results. However, the user is encouraged to carefully inspect a wider range of hits beyond the ones highlighted in this column for possible functional associations with the query.

Mouseover of hit names displays the description of the protein and the amino acid positions of the given domain with the protein. Clicking on a hit name redirects to the corresponding entry for this specific sequence in the PFAM database, which allows for a deeper inspection of this individual domain of a given protein, the PFAM domain family that it belongs to, and its biological function. The heatmap in shades of blue on the right displays normalized similarity scores for a given domain across all genomes in the chosen evolutionary range. Taxonomic tree on top is derived from NCBI Taxonomy database (31,32) and helps connecting the patterns of conservation to specific taxonomic groups. Mouseover of the heatmap shows more detailed information about each element of the heatmap: the name of domain family, the full name of the species, and the corresponding normalized BLAST score between the hit domain in this species and the query domain. As a new functionality of DEPCOD compared to PhyloGene (7), three additional columns on the left display known protein-protein interactions between the query and the hit, according to BioGrid (23) (‘BioGrid Interaction’) and Hu.Map 2.0 (24) (‘HuMap Interaction’), as well as the presence of a shared protein complex (‘HuMap Complex’). The numerical version of the score heatmap can be downloaded as a tab-delimited text file or Excel spreadsheet by clicking ‘Download’ button on top of the page.

As another new functionality in DEPCOD, the enrichment of functional protein categories and pathways (Figure 1B) is displayed on the bottom of the webpage below the main heatmap, in three tabs showing top enriched pathways from GO (25,26), KEGG (27,28) and Reactome databases (29) as bar plots of statistical significance (Benjamini-Hochberg FDR in log scale). This section can also be quickly accessed from the top of the webpage using button ‘Gene Set Enrichment’.

The URL for the whole result webpage can be quickly copied for the user's records or sharing using button ‘Share’ in the top section of the page.

### Phylogenetic profile similarities detected between domains but not between full protein sequences

In the example of DEPCOD output shown in Figure 1A, submitting a protein kinase domain of MAP2K6 protein, a member of MAPK signalling pathway, as a query results in the detection of phylogenetic profile similarities to many other functionally related domains. These domains include kinase domain homologs but also many non-homologous domains that belong to the members of MAPK pathway and related signaling pathways (Figure 1B): RAS, RHO, TCR, VEGF signaling, regulation of cell cycle, etc.

The focus of DEPCOD on individual domains improves phylogenetic profiling by preventing possible effects produced by the changes of domain architecture that are frequent in the evolution of multidomain proteins. This focus allows DEPCOD to detect profile similarities between functionally associated domains that are challenging to detect from whole-protein sequences. As an example, the kinesin domain of STARD9 protein is a part of multidomain protein architecture that varies among protein homologs across different species. As shown in Figure 1C, human STARD9 protein sequence includes a kinesin domain at the N-terminus followed by two other PFAM domains, FHA and START, although the corresponding proteins in other species, especially in more divergent fly and worm genomes, share the N-terminal kinesin domain but have different domain arrangements in the C-terminal part.

This variability of domain architecture creates a challenge for the accurate assessment of sequence similarity between whole-protein sequences, often resulting in weaker phylogenetic profiles across multiple species and therefore weaker profile similarity between whole proteins. As a result, some of the similarities between phylogenetic profiles detected at the domain level (Supplementary Figure S2) are missed at the level of whole protein sequences. For example, RAN and EIF4A3 proteins functionally related to STARD9 could not be detected by phylogenetic profiling at the whole protein level with STARD9 protein as a query. Implemented in our previous method PhyloGene (7), these whole-protein profiles of STARD9, EIF4A3 and RAN do not share a strong similarity (Figure 1D, left heatmap). By contrast, DEPCOD’s focus on individual domains facilitates the detection of local sequence similarity, resulting in clearer phylogenetic profiles and stronger similarities between these, often non-homologous domains: kinesin domain of STARD9, Helicase C and DEAD domains of EIF4A3, and Ras domain of RAN (Figure 1D, right heatmap). These proteins are functionally related through their known roles in the regulation of microtubule assembly in mitotic spindle. STARD9 is a centrosomal protein involved in spindle assembly by enabling microtubule binding and microtubule motor activity through its kinesin domain (33,34); EIF4A3 is a core component of the exon junction complex (EJC) which is also localized to centrosomes and involved in microtubule interactions during mitosis (35,36); whereas RAN GTPase localizes to chromosomes during mitosis and is a central regulator of chromatin-driven microtubule polymerization in mitotic spindle assembly (37).

The analysis of phylogenetic profiles based on only eukaryotic species allows for a higher-resolution survey of correlations based on eukaryotic evolution, especially for the domains whose homologs are restricted to eukaryotes. However, expanding the range of species from eukaryotes to 506 species across the tree of life allows for the detection of many additional profile correlations for the domains with prokaryotic homologs. As an example, eukaryotic phylogenetic profiles of RNA_pol_Rpb1_1 domain of human POLR3A, RNA_pol_Rpb2_1 domain of POLR1B, and RIO1 domain of RIOK1 protein had only modest similarity (Pearson R < 0.5) to the profile of tRNA-synt_1 of LARS protein as a query (Figure 1E). In fact, profile correlations between most members of this functionally related group were modest. However, their profiles based on the whole tree of life had much stronger correlations to the query and to each other (Pearson *R* > 0.75, Figure 1E). These proteins are functionally related through their known roles in ribosomal function. LARS is leucine tRNA synthase, POLR3A is a subunit of specialized RNA polymerase III responsible for the synthesis of tRNAs, ribosomal 5S rRNA, and other small RNAs, POLR1B is a subunit of RNA polymerase I responsible for the production of ribosomal RNAs, whereas RIOK1 (RIO kinase 1) plays essential role in maturation of 40S ribosomal subunits (38). Phylogenetic profiles of all four domains across the whole tree of life (Supplementary Figure S3) share a strong similarity beyond eukaryotes: they have similar levels of conservation among their homologs in *Archaea* and a drop of conservation signal in *Bacteria*.

We performed a comprehensive evaluation of detection accuracy for the modes of DEPCOD that use phylogenetic profiles calculated (a) among eukaryotes only and (b) across the whole tree of life, and further compared these DEPCOD modes to the PhyloGene approach (7) that uses whole-protein profiles. Using shared functional pathways as a reference, we generated precision/recall plots for all three approaches, based on top 100 hits with Pearson *R* > 0.6 for all PFAM domains in human genome. Figure 1F shows precision/recall plots using shared KEGG pathway as a reference of functional association between proteins. Supplementary Figures S4A, B show similar plots based on GO Biological Processes and Reactome databases as references. These plots suggest two important observations. First, domain-based approach implemented in DEPCOD has a higher detection accuracy compared to the whole-protein approach implemented in PhyloGene. Second, DEPCOD phylogenetic profiles based on the whole tree of life provide a higher overall accuracy of detection compared to the profiles based on eukaryotic species only.

### Groups of similar phylogenetic profiles reveal specific domain functions and evolutionary history

**Figure 2.**
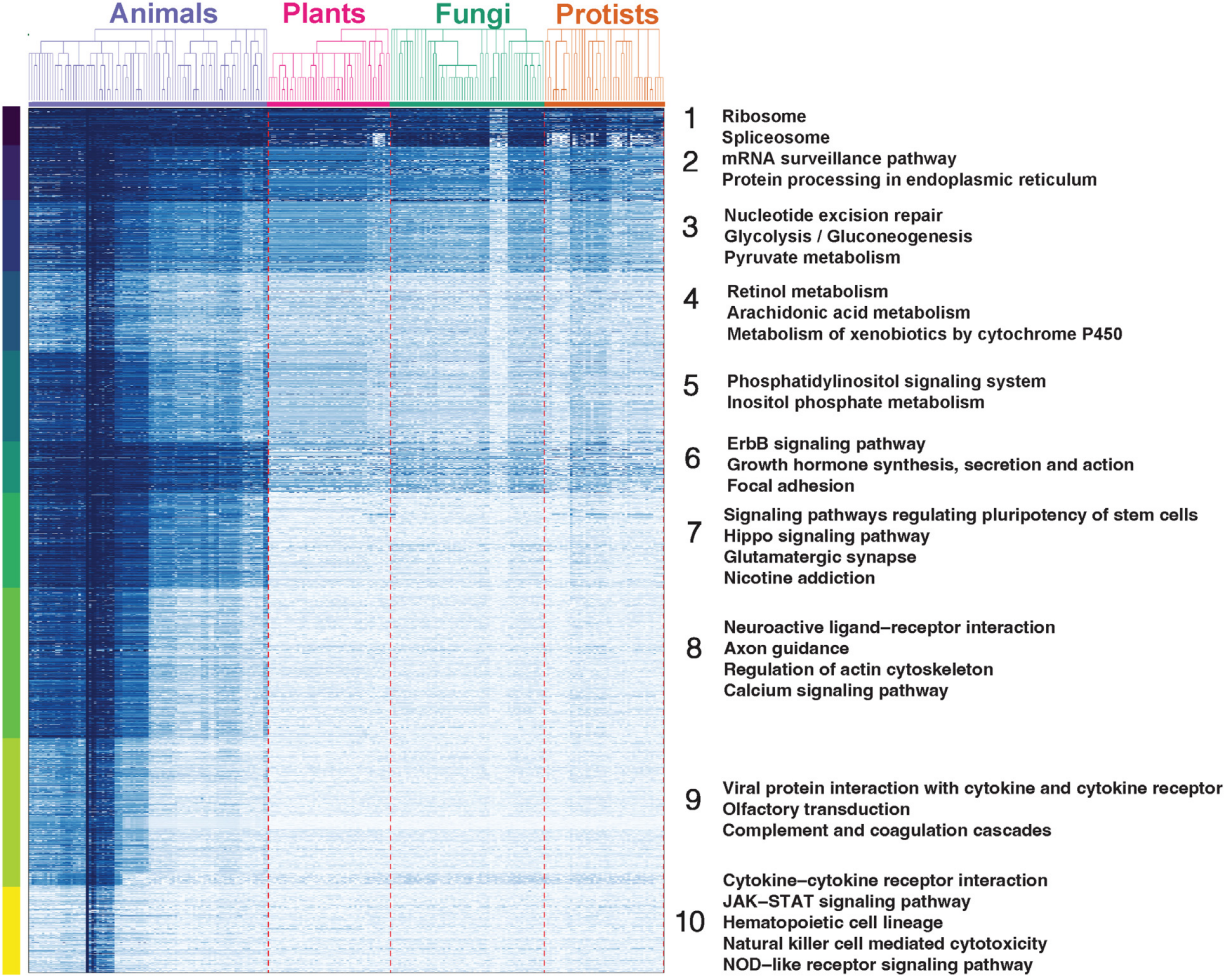
Comprehensive analysis of DEPCOD phylogenetic profiles among all human protein domains reveals functionally related clusters with specific patterns of evolutionary history. Heatmap of phylogenetic profiles for a subset of all human domains. Rows, domains clustered by the similarity of their phylogenetic profiles (hues of blue) across eukaryotic species (columns, with taxonomic tree of genomes shown on top). Functional protein categories enriched in these clusters are indicated on the right.

On genome-wide scale, similarities between DEPCOD phylogenetic profiles are associated with both specific protein functions and specific evolutionary patterns of sequence conservation among protein domains. Figure 2 shows the heatmap of phylogenetic profiles for a large subset of all human domains clustered by the profile similarity (Pearson correlation coefficient). Each of the resulting large domain groups shares a pattern of phylogenetic conservation across eukaryotes regardless of homology between these domains. Based on the KEGG pathway enrichment analysis using the EnrichR method (39,40), these groups are enriched in specific protein functions, revealing associations between these functions and evolutionary history (Figure 2, Supplementary Table S1). Some of these associations are well known, whereas others are less expected. For example, clusters 1 and 2 (Figure 2, top of the heatmap) include domains with strong conservation patterns across all eukaryotic species and are enriched in functional KEGG categories related to ribosomal, spliceosomal, and other functional categories that are universally and strongly conserved among eukaryotes. These profile correlations sometimes correspond to coincidental but functionally unrelated major evolutionary innovations that occurred during the early rise of eukaryotes. However, even among domains whose homologs are present across all eukaryotes, detailed analysis of phylogenetic profiles often can provide new insights into distinct quantitative subtypes of conservation patterns and their association with distinct functions. As an example, clusters 3, 4 and 5 include domains that are also present across all eukaryotes but show a stronger contrast between sequence conservation among animals and other eukaryotic clades (Figure 2). Interestingly, these clusters correspond to other basic cellular functions that are ubiquitous among eukaryotic species, but these domain sequences are apparently less conserved outside the animal kingdom. These functions are often associated with fundamental metabolic (glycolysis, pyruvate metabolism, metabolism of xenobiotics by cytochrome P450, etc.) or signaling functions (phosphatydilinositol signaling etc.), suggesting a stronger evolutionary variation for these domains than for the domains involved in transcription, splicing, and translation (clusters 1 and 2).

Domains with animal-specific distribution (clusters 6–8) are enriched in well-known animal-specific functions associated with the nervous system (neurotransmission, axon guidance, neuroactive ligand-receptor interaction) and animal development (stem cell pluripotency, growth hormone synthesis), but also in less expected functions such as focal adhesion, regulation of actin cytoskeleton, and calcium signaling, suggesting animal-specific evolutionary innovations in these more general eukaryotic pathways. Finally, mammalian-specific domains (cluster 9) are enriched in blood and adaptive immunity functions (hematopoietic cell lineage, cytokine-cytokine receptor interaction, natural killer cytotoxicity, JAK/STAT and NOD—like receptor signaling), consistent with the known expansion and specialization of these functions in mammals. These similarities of phylogenetic conservation patterns among human domains with specific biological functions suggest that DEPCOD is a valuable tool for both detailed analyses of domain evolution and the prediction of potential functional associations between protein domains.

## CONCLUSIONS

As a further development of the normalized phylogenetic profiling approach initially implemented in our PhyloGene webserver, DEPCOD focuses on sequence conservation patterns of protein domains as mobile evolutionary units. This more focused analysis allows the detection of similarities between phylogenetic profiles and prediction of potential functional associations between non-homologous domains beyond the capabilities of the whole-protein approach. Importantly, the new DEPCOD server provides various types of additional information to help the user inspect biological functions of the detected domains, their known physical interactions and protein complexes shared with the query, and aid in detailed manual analyses of possible sources and evolutionary relevance of the detected conservation similarities. As a result, DEPCOD is an informative and efficient web server for the analyses of evolutionary and functional associations between protein domains.

In the future, we plan to focus on a few methodological directions to extend and improve DEPCOD. First, we will develop a separate mode for the detection of anti-correlations between phylogenetic profiles. These anti-correlations correspond to mutual exclusion or, at a more gradual scale, opposite patterns of sequence conservation among homologs of the two compared domains across the range of species, which may suggest redundant or otherwise related functions of the two domains. Second, to fully leverage the rich evolutionary information among prokaryotes, in addition to the modes of ‘Eukaryotes’ and ‘All clades’, we plan to introduce the modes of ‘Prokaryotes’ (phylogenetic profiles based on *Bacteria* and *Archaea*) and ‘Bacteria’ (phylogenetic profiles based on *Bacteria* only). In these two modes, we will add new prokaryotic organisms as query species. Third, we will further extend the set of organisms available as query species in all DEPCOD modes and expand the number of organisms used in phylogenetic profiles.

## DATA AVAILABILITY

DEPCOD is freely available at http://genetics.mgh.harvard.edu/DEPCOD.

## Supplementary Material

gkac349_Supplemental_Files
