# Supplementary material for: DEPCOD: a tool to detect and visualize co-evolution of protein domains
Source: Nucleic Acids Res. 2022 May 10;50(W1):W246–53. doi: 10.1093/nar/gkac349 (PMC9252791; doi:10.1093/nar/gkac349)
Supplement: gkac349_Supplemental_Files [file gkac349_supplemental_files.zip › DEPCOD_webserver_supplement.pdf]

DEPCOD: a tool to detect and visualize co-evolution of protein domains

Fei Ji[1,2 ‡], Gracia Bonilla [1,2 ‡], Rustem Krykbayev[1], Gary Ruvkun[1,2], Yuval Tabach[3], and Ruslan I. Sadreyev[1,4 *]

[1] Department of Molecular Biology, Massachusetts General Hospital, Boston, MA, USA

[2] Department of Genetics, Harvard Medical School, Boston, MA, USA

[3] Department of Developmental Biology and Cancer Research, Faculty of Medicine, The Hebrew University of Jerusalem, Ein Kerem, 9112102 Israel

[4] Department of Pathology, Massachusetts General Hospital and Harvard Medical School, Boston, MA, USA

[‡] The authors wish it to be known that, in their opinion, the first two authors should be regarded as joint First Authors.

SUPPLEMENTARY MATERIALS

Supplementary Table 1 (Excel file KEGG_enrichment_in_domain_clusters.xls, supplement to Figure 2).

Statistical significance of enrichment (adjusted P-value estimates by EnrichR) of KEGG pathways enriched in clusters of human protein domains grouped by the similarity of their phylogenetic profiles. Rows: KEGG pathways; columns: clusters of domains shown in Figure 2 (cluster 1-10).

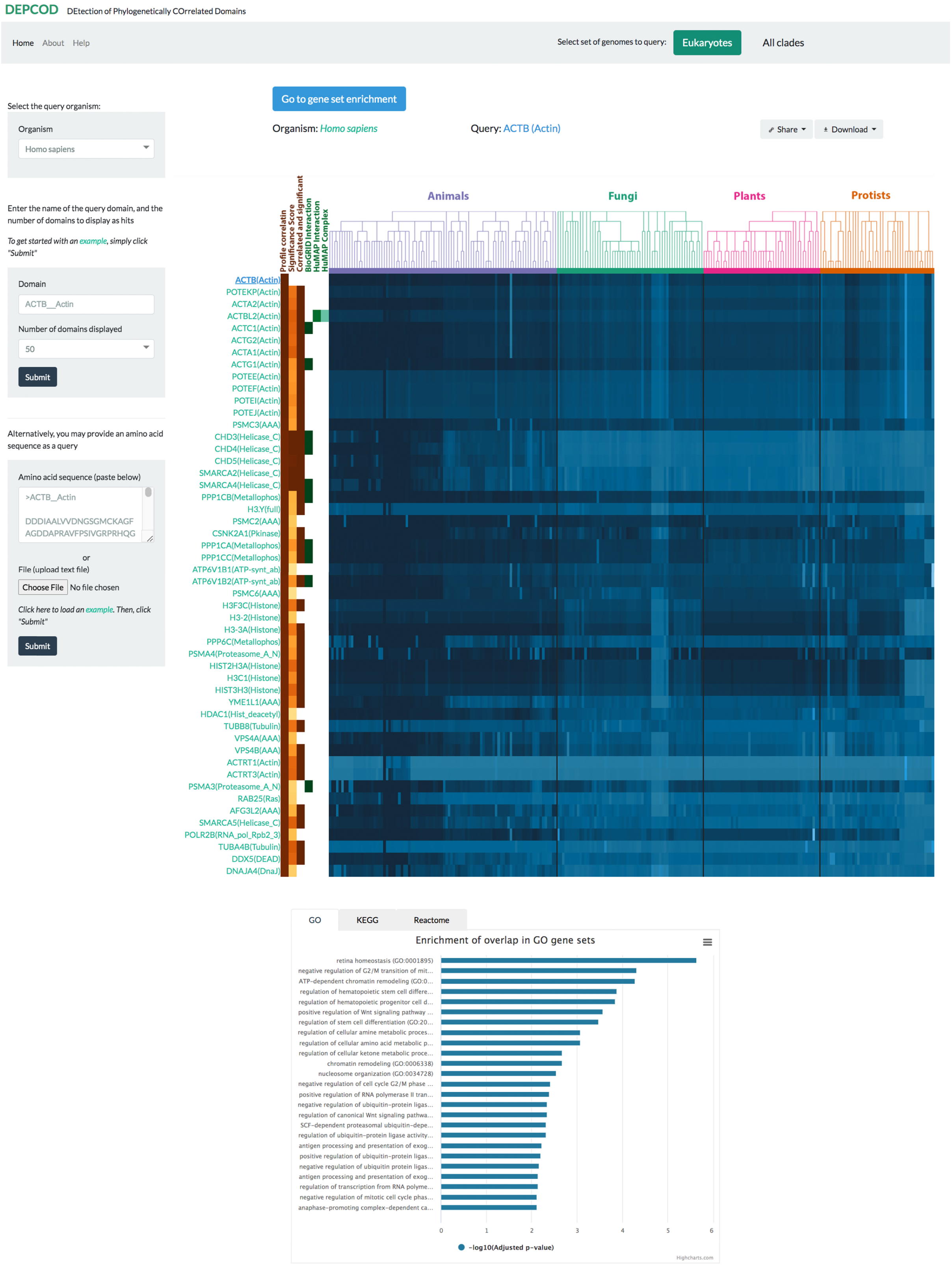

Supplementary Figure 1. A screenshot of DEPCOD webpage with the areas for the user-defined input and for the display of DEPCOD search results: the heatmap of top phylogenetic profiles similar to the query domain (Actin domain of human ACTB protein), functional enrichment analyses among the top hits, and various additional information.

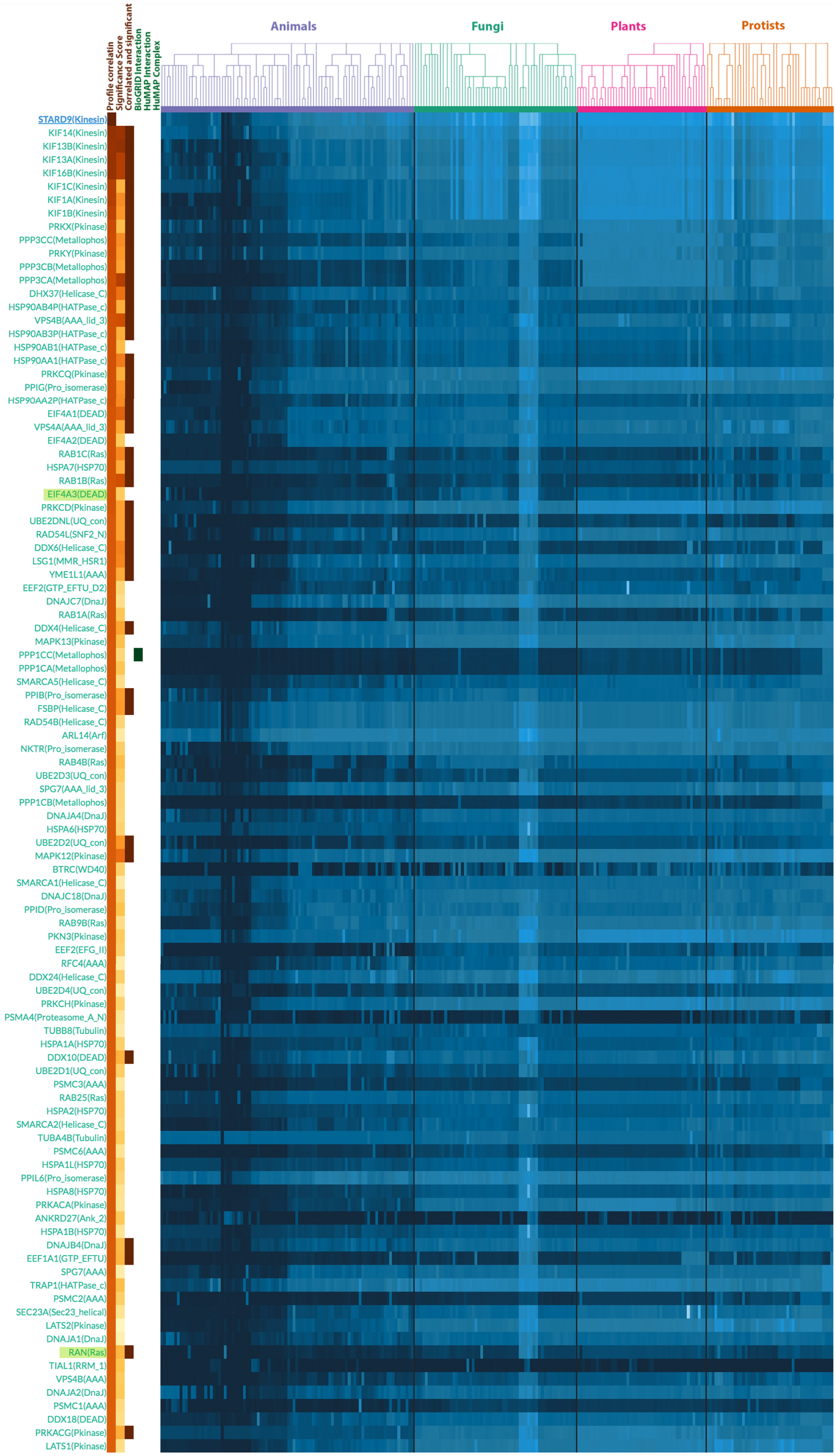

Supplementary Figure 2. The heatmap of top eukaryotic phylogenetic profiles most similar to that of Kinesin domain from human STARD9 protein as a query. Rows, top human domain hits, with query

domain on top. Columns, individual eukaryotic species, with the taxonomic tree shown on top. Hues of blue indicate normalized sequence similarity scores across all species to the human domain. EIF4A3 and RAN domains (highlighted) were detected as hits by DEPCOD but not by the whole-protein approach implemented in the previous PhyloGene server (see Fig. 1C,D).

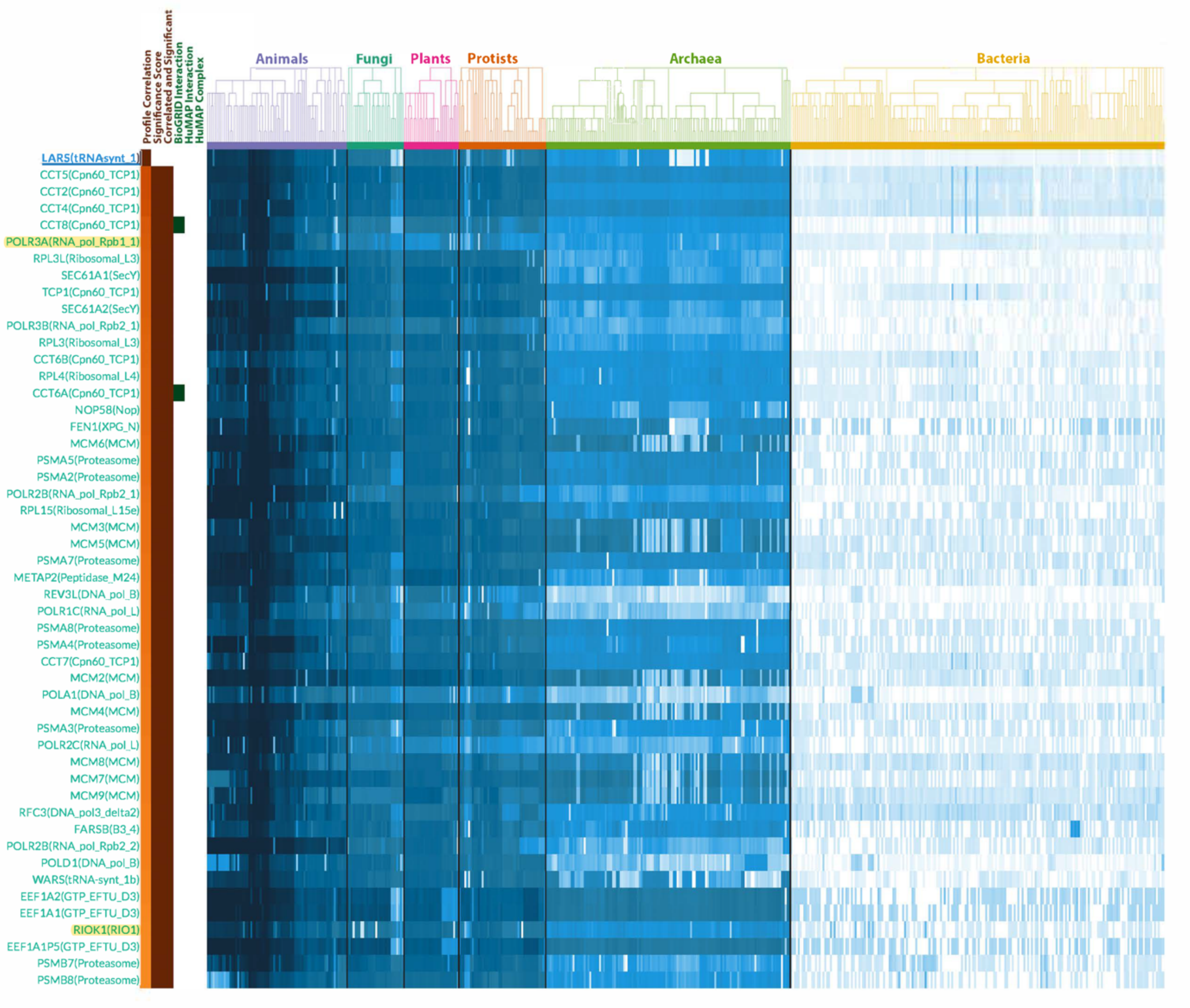

Supplementary Figure 3. The heatmap of top phylogenetic profiles generated across the whole tree of life that show the strongest correlation to the profile of tRNA synthase 1 domain of human LARS protein as a query. The highlighted domains had a strong correlation to the query when profiles were based on the whole tree of life but were not detected using profiles based on eukaryotes only (see also Fig. 1E). Close homologs of these domains are present outside eukaryotes in most of *Archaea* but much lesss in *Bacteria*. Rows, top human domain hits, with query domain on top. Columns, individual eukaryotic species, with the taxonomic tree shown on top. Hues of blue indicate normalized sequence similarity scores across all species to the human domain.

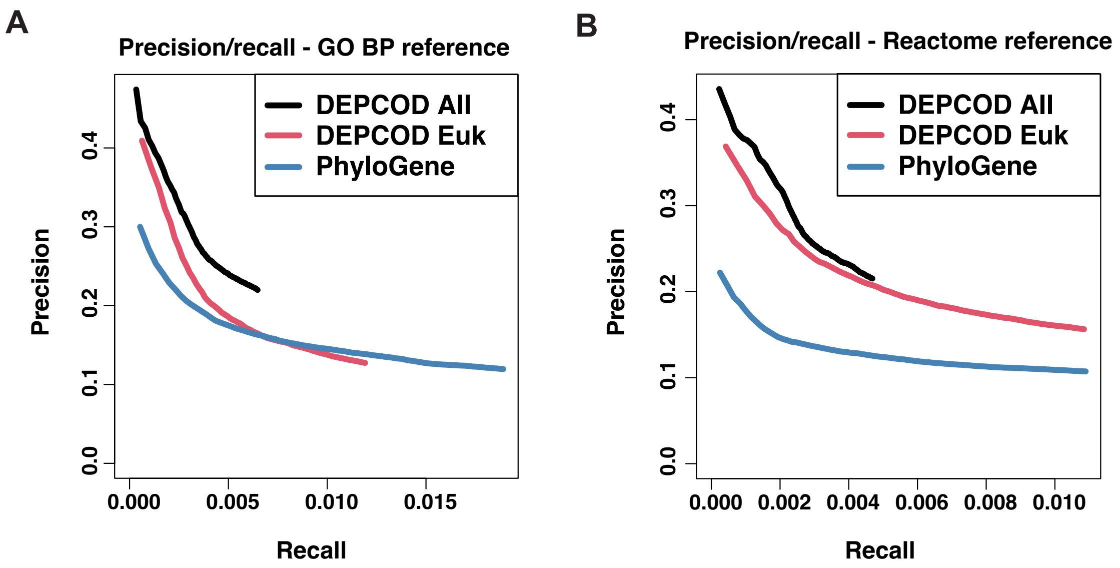

Supplementary Figure 4. Precision/recall curves comparing the accuracy of detecting functional protein associations using phylogenetic profiles based on whole proteins (PhyloGene) and on protein domains (DEPCOD). DEPCOD mode with phylogenetic profiles based on the whole tree of life (DEPCOD All) has a higher accuracy than the mode based on eukaryotes only (DEPCOD Euk). The definition of true positive hits was based on sharing the same functional category with the query, using GO Biological Processes (A) or Reactome database (B) as references. See also Fig. 1F.
